# Design and Methodology of a Pilot Randomized Controlled Trial of Transcranial Direct Current Stimulation in Acute Middle Cerebral Artery Stroke (STICA)

**DOI:** 10.3389/fneur.2018.00816

**Published:** 2018-10-09

**Authors:** Estelle Pruvost-Robieux, David Calvet, Wagih Ben Hassen, Guillaume Turc, Angela Marchi, Nicolas Mélé, Pierre Seners, Catherine Oppenheim, Jean-Claude Baron, Jean-Louis Mas, Martine Gavaret

**Affiliations:** ^1^Department of Neurophysiology, Sainte-Anne Hospital, Paris, France; ^2^Faculty of Medicine, Paris Descartes University, Paris, France; ^3^INSERM UMR S894, Paris, France; ^4^Department of Neurology, Sainte-Anne Hospital, Paris, France; ^5^Department of Neuroradiology, Sainte-Anne Hospital, Paris, France

**Keywords:** acute ischemic stroke, functional outcome, tDCS, cortical spreading depolarization, peri-infarct depolarizations

## Abstract

**Background:** Stroke is a major cause of death and disability worldwide. The related burden is expected to further increase due to aging populations, calling for more efficient treatment. Ischemic stroke results from a focal reduction in cerebral blood flow due to the sudden occlusion of a brain artery. Ischemic brain injury results from a sequence of pathophysiological events that evolve over time and space. This cascade includes excitotoxicity and peri-infarct depolarizations (PIDs). Focal impairment of cerebral blood flow restricts the delivery of energetics substrates and impairs ionic gradients. Membrane potential is eventually lost, and neurons depolarize. Although recanalization therapies target the ischemic penumbra, they can only rescue the penumbra still present at the time of reperfusion. A promising novel approach is to “freeze” the penumbra until reperfusion occurs. Transcranial direct current stimulation (tDCS) is a non-invasive method of neuromodulation. Based on preclinical evidence, we propose to test the penumbra freezing concept in a clinical phase IIa trial assessing whether cathodal tDCS—shown in rodents to reduce infarction volume—prevents early infarct growth in human acute Middle Cerebral Artery (MCA) stroke, in adjunction to conventional revascularization methods.

**Methods:** This is a monocentric randomized, double-blind, and placebo-controlled trial performed in patients with acute MCA stroke eligible to revascularization procedures. Primary outcome is infarct volume growth on diffusion weighted imaging (DWI) at day 1 relative to baseline. Secondary outcomes include safety and clinical efficacy.

**Significance:** Results from this clinical trial are expected to provide rationale for a phase III study.

**Clinical trial registration**—EUDRACT: 2016-A00160-51

## Introduction

Ischemic stroke is a main etiology of death or severe disability. It affects over 13.5 million of people worldwide annually. Ischemic stroke results from a focal reduction in cerebral blood flow due to the sudden occlusion of a brain artery. In the first hours after ischemic stroke brain imaging can distinguish an ischemic core (necrosis of neurons) and a penumbral area where hypoperfusion is mild to moderate. Neurologic disability is the consequence of both the ischemic core and the penumbra. In the penumbra, with energy depletion, membrane potential is eventually lost, neurons and glia depolarize, and cell necrosis ensues. Current opportunities to salvage the ischemic penumbra are based on early recanalization of the occluded artery allowing early reperfusion of ischemic tissue. Three strategies are available: IV thrombolysis with recombinant tissue plasminogen activator (rtPA), endovascular thrombectomy (EVT), or the combination of IV thrombolysis and EVT (1–9). However, despite these procedures, only 46% of patients have mild disability at 3 months (10). To improve functional outcome, numerous neuroprotective strategies appeared promising in preclinical models. However, so far they have failed in clinical trials (11, 12).

A promising novel strategy for neuroprotection is to “freeze” the ischemic penumbra until reperfusion occurs (i.e., prevent further demise of the penumbra until reperfusion) (13). Among the factors that may affect the course of the penumbra, Leão in 1947 first described cortical spreading depression (CSD) characterized by a spreading depolarization of neurons and glial cells associated with ion homeostasis breakdown (14). In stroke, CSDs propagate as peri-infarct depolarizations (PIDs) across the cerebral cortex from the ischemic core to the penumbra, and are associated with infarct growth (15–17).

Transcranial direct current stimulation (tDCS) is a non-invasive brain neuromodulation technique that uses direct electrical currents to stimulate specific parts of the brain. A constant, low intensity current is passed through two electrodes placed over the head, which modulates neuronal activity. Polarizing currents are produced and able to cross the skull and induce sustained changes in membrane potential and excitability of cortical cells. tDCS may affect neuronal and glial transmembrane potential (18–20). The widespread opinion is that cathodal tDCS reduces cortical excitability whereas anodal stimulation increases it (by hyperpolarization and depolarization, respectively) (21). Cathodal tDCS blocked CSD initiation in a rat model where CSDs are elicited by pricking the cortex with a needle (22). Moreover, three studies in ischemic stroke rodent models demonstrated a neuroprotective effect of cathodal tDCS with a significantly reduced infarct size relative to controls (see section Discussion for details) (23–25). Only a few tDCS studies have been conducted in humans at the early stage of MCA stroke. However, these studies were performed with a different objective: to promote early motor recovery, using anodal tDCS stimulation facing the stroke area (26–29). In these studies, no adverse effect was described.

Based on preclinical studies, we hypothesized that, probably via blocking CSDs and PIDs, cathodal tDCS may limit infarct growth in human acute MCA stroke.

We present here the design of the STICA trial: “tDCS in acute human ischemic stroke, a pilot, prospective, double-blind, placebo-controlled randomized clinical trial.”

## Study design and methods

### Subjects eligibility, ethical considerations, and recruitment

#### Inclusion and exclusion criteria

Initial inclusion criteria were adult patients (age over 18 years-old) with acute ischemic stroke in MCA territory (no lacunar stroke) proved by MRI; with time from stroke onset to treatment minor than 4:30 h; National Institutes of Health Stroke Scale (NIHSS) between 4 and 25; and eligible to receive IV thrombolysis with rtPA. Two amendments were subsequently adopted in order to include all patients with acute ischemic stroke in the MCA territory eligible to a revascularization procedure (irrespective of the revascularization procedure—IV thrombolysis and/or EVT—and irrespective of the NIHSS score). Exclusion criteria are contraindications to MRI, forehead skin injuries, history of intracranial surgery (because it implies skull breach which could modify tDCS current distribution), consciousness impairment (in order to uniformly assess tDCS safety between participants), and pregnancy “known at the patient's admission” (for ethical considerations).

#### Ethical considerations and dissemination

All patients provide written informed consent in accordance with the Declaration of Helsinki before inclusion in this study if they can provide it. Otherwise, their relatives are asked for consent. If no relative is available, the patient can be included using an “emergency procedure.” If consent is given by relatives or if the patient is included using the “emergency procedure,” a complementary consent has to be completed by the patient as soon as possible. The study protocol was approved by the local ethics committee (2016-A00160-51, ref CPP 3373) and registered at EUDRACT: 2016-A00160-51.

The STICA protocol is intended to evaluate the potential of cathodal tDCS to limit ischemic damage in acute human MCA stroke, as an adjunct to standard-of-care revascularization strategies. The results from this study will be disseminated to healthcare professionals via publication in a peer-reviewed journal. Moreover, we intend to present the findings in international stroke, neurophysiology, and neuromodulation conferences.

### Trial design, randomization and intervention

#### Trial design

Our study is an ongoing monocentric prospective double-blind and placebo-controlled clinical phase IIa trial with randomized treatment-group allocation, according to CONSORT statements (30). tDCS plus usual care (thrombolysis ± thrombectomy) is compared to sham-tDCS plus usual care (control group) in patients with acute MCA stroke. Patients are being recruited in Sainte Anne's hospital Stroke unit since December 2016. Complete recruitment is expected in June 2019.

In our institution, endovascular treatment is considered in acute middle cerebral artery (MCA) stroke in the following situations: if time of stroke onset is <6 h, and if there is a “proximal” arterial occlusion (internal carotid, M1 and proximal section of part M2 of MCA). Until the 27th of June 2018, endovascular treatment was not used if time of stroke onset was beyond 6 h. Since this date, if last-seen-well time is between 6 and 24 h, endovascular treatment is performed if the patient meets the criteria of the DEFUSE 3 or DAWN trial criteria (31, 32).

All subjects undergo MRI scanning at baseline, just before enrollment. MRI protocol includes Diffusion Weighted Imaging (DWI), Fluid Attenuated Inversion Recovery (FLAIR), 3D-Time-Of-Flight imaging, and Perfusion-Weighted-Imaging (PWI) whenever possible. PWI was not included routinely because it is not required to assess the primary outcome of our study. Moreover, some severely affected patients with hyper-acute stroke or speech disturbances can be agitated or unstable and the MR exam occasionally has to be interrupted before PWI acquisition. If they meet inclusion criteria, subjects are randomized to receive either cathodal or sham tDCS in adjunction to a recanalization procedure, in a complete double-blind fashion. All participants undergo a second MRI scanning at day 1 (with DWI for primary outcome assessment) and clinical examination at day 7 (or discharge if earlier). Follow-up is obtained at 3 months by phone interview by one investigator (blinded to randomization) in order to assess the modified Rankin Scale (33).

#### Randomization

All patients are randomly assigned to receive either cathodal or sham-tDCS. The randomization list was previously established with a biostatistician (using the block method), allocating a different 5-digit code to each patient included, in a sealed envelope. All five-digit codes are pre-programmed into the tDCS device to deliver either active or sham tDCS and the randomization list was transmitted to the biostatistician to maintain the blinding of all experimenters. At the time of inclusion, just after the MRI scanning and before revascularization treatment whenever possible (otherwise, during the revascularization procedure), the experimenter opens the sealed envelope corresponding to the number of the patient being included and discovers its 5-digit code.

#### Intervention

**Figure 1:**
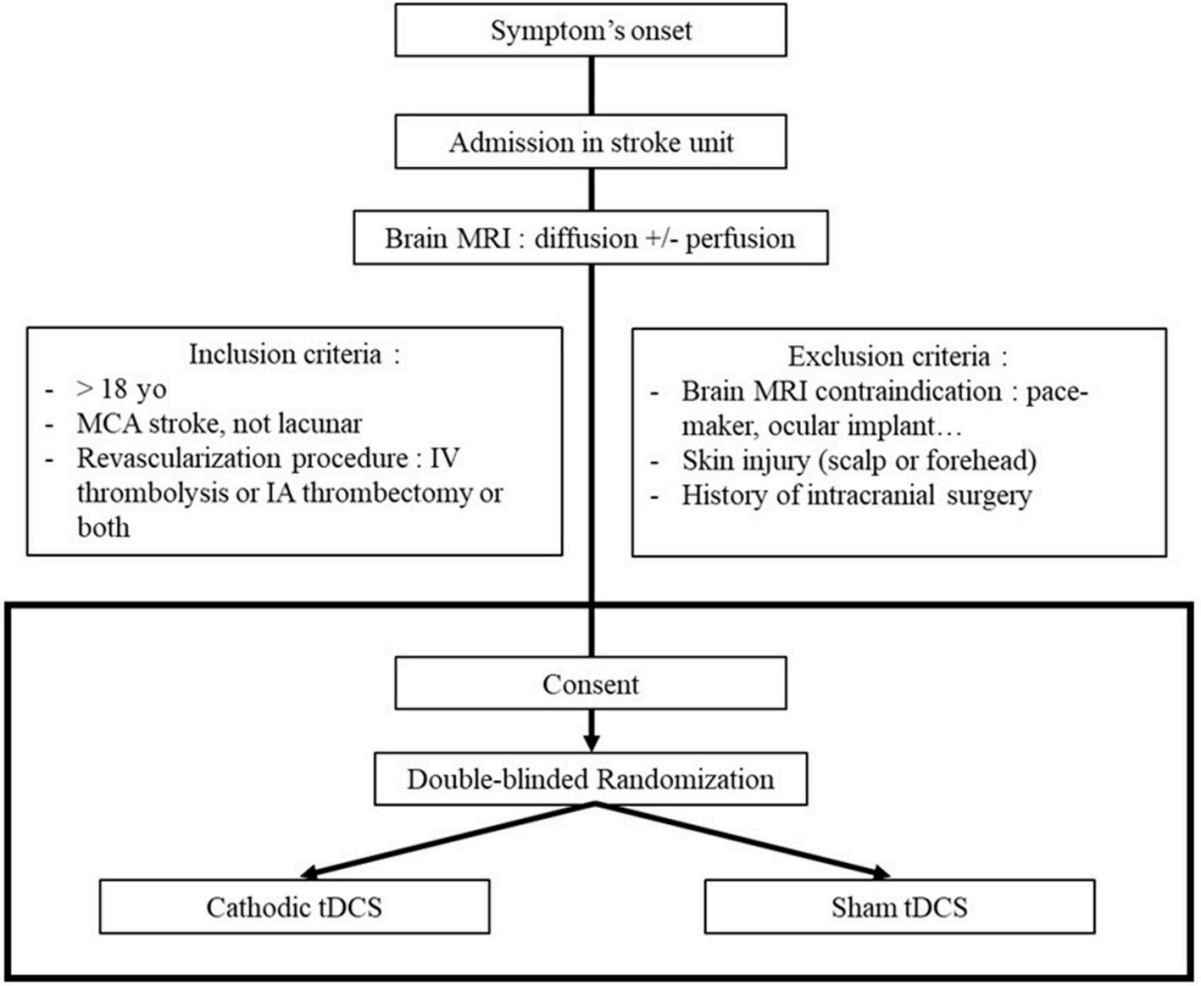
Study design.

**Figure 2:**
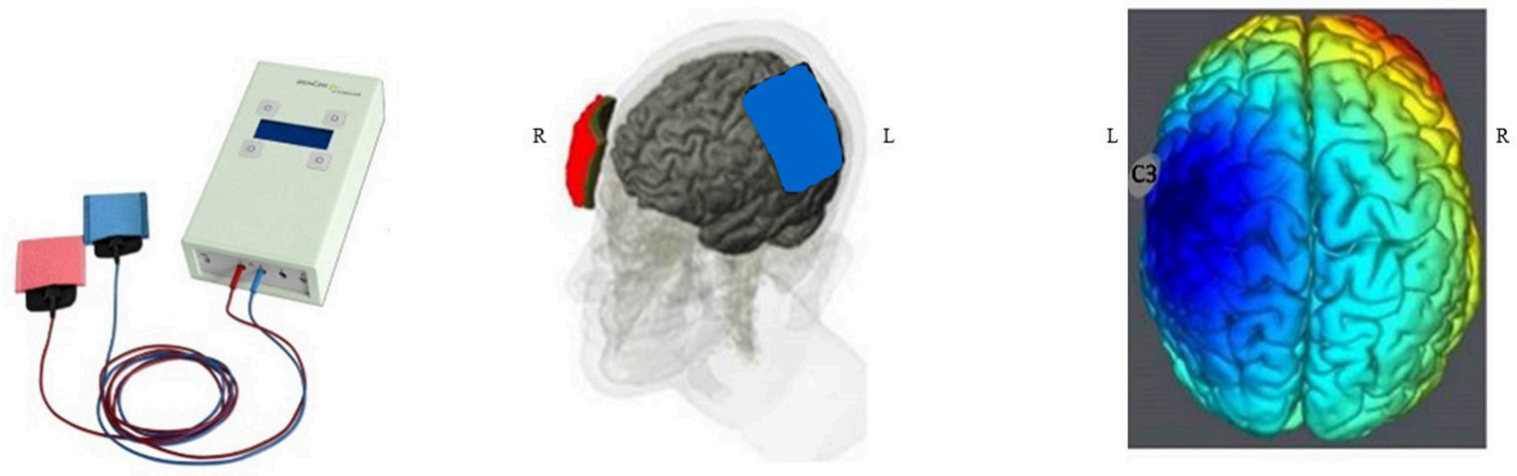
tDCS device and tDCS electrode positioning. In case of left MCA stroke, in order to hyperpolarize the ischemic penumbra, the cathode (in blue) faces the ischemic injury (C3, 10–20 system) and the anode (in red) faces the contralateral supra-orbitary area.

**Figure 3:**
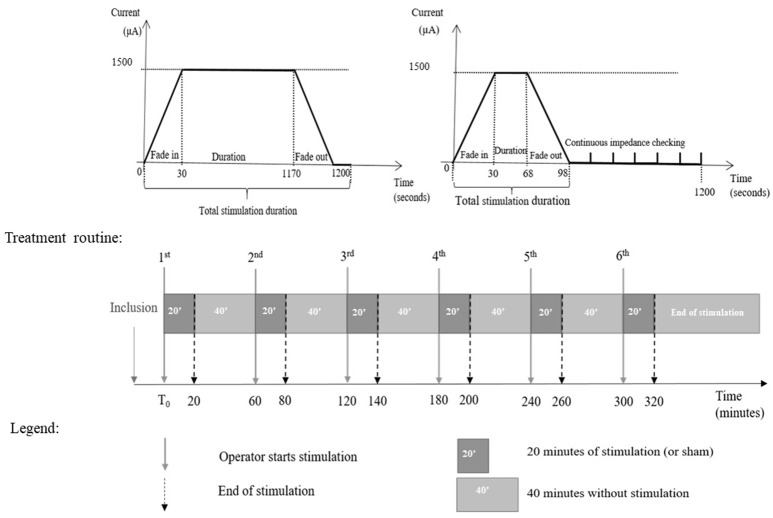
Timelines of active tDCS (left) and sham tDCS (right), and treatment protocol.

**Figure 4:**
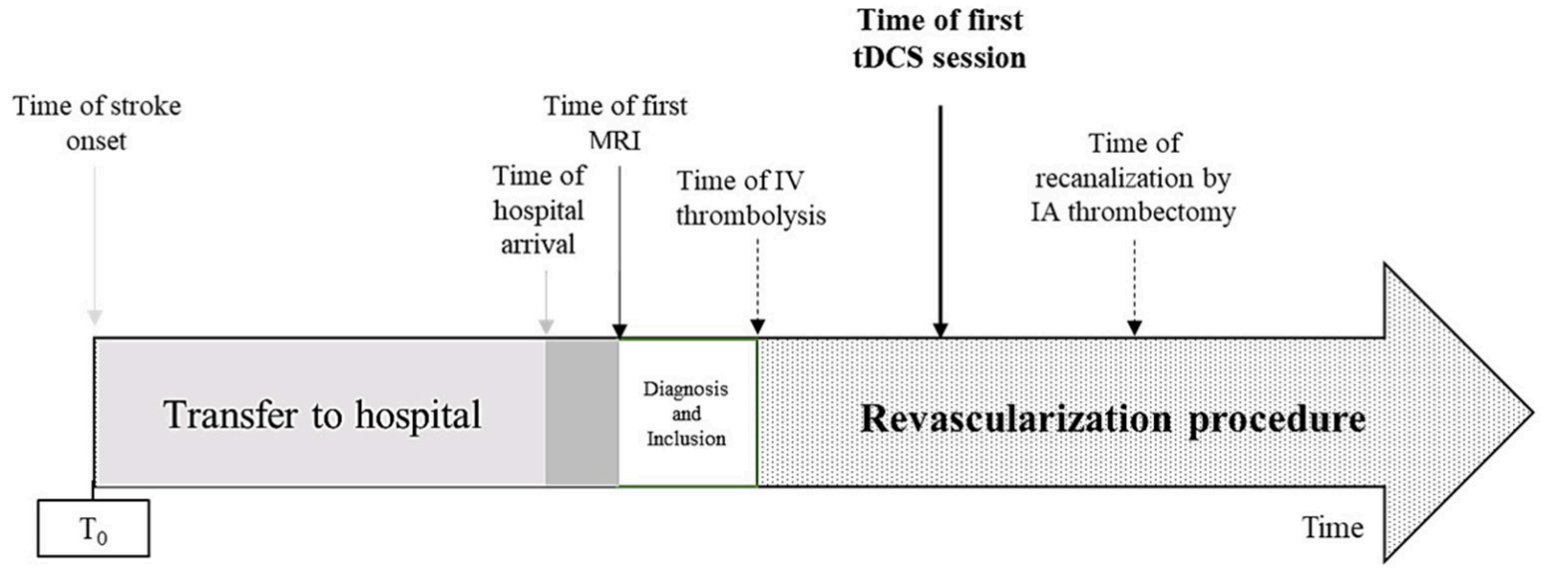
Timeline of procedures. IA, intra-arterial; IV, intravenous.

The intervention consists in cathodal tDCS by the DC-STIMULATOR PLUS (neuroConn, Ilmenau, Germany), a CE-certified medical device, programmed to deliver either active or sham-tDCS according to a randomization 5-digit code. tDCS or sham is started as soon as possible after the inclusion, in the stroke unit or in the angiosuite during thrombectomy, in addition to reperfusion therapies. The investigator sets the cathode facing the ischemic injury (C3 or C4 according to the 10–20 system) and the anode facing the contralateral supra-orbital area (Figure 1). Then, the experimenter enters the 5-digit randomization code in the device, which blindly determines whether cathodal or sham stimulation is to be delivered, without the experimenter knowing which. Delivery of stimulation is for 20 min. Over a period of 6 h, the experimenter restarts the device every hour with the same 5-digit randomization code for another 20 min. The device delivers 1.5 mA (with 30 s for ramping up and ramping down) of cathodal direct current through the two sponge coated electrodes (electrodes size: 5 × 7 cm^2^; Figure 2). Impedance quality is obtained using saline solution and conductive gel (Elefix paste®). The device automatically checks impedance values during all tDCS sessions. In the sham condition, the device delivers 98 s of current including 30 s for ramping at the beginning and end of each session, in order to keep the blinding procedure (Figure 3). Indeed, usually, subjects feel discomfort under the electrodes during only the first few seconds or minutes of tDCS stimulation (18, 34). The experimenter notes every significant event: time of stroke onset, time of hospital arrival, time of first MRI, time of IV thrombolysis, and/or endovascular recanalization, time of each tDCS session (Figure 4).

### Safety aspects

During the procedure, potential tDCS adverse events are checked every hour: itching, irritation, or rash, burning feelings under the electrodes, headaches, nausea, and dizziness. All these minor side events are reversible when the tDCS stops (35). To minimize these risks, a dedicated experimenter is present during each tDCS stimulation session. This experimenter asks the patient about any side-effect or discomfort and notes these data each hour. The tDCS procedure is stopped in case of adverse events.

In order to minimize the risk of rash or burning feelings, the experimenter adds saline solution and conductive gel (Elefix® paste) every hour if necessary, according to impedance values. High impedances are indeed a contributing factor for skin lesions (35). Moreover, the device automatically stops in case of impedance value superior to 15 kΩ. An independent data and safety monitoring board analyzes STICA safety data after the recruitment of 25 patients.

### Outcomes

#### Primary outcome

The primary outcome is infarct growth (IG, in mm) determined using the MRI obtained at admission (DWI_1_) and 1 day later (DWI_2_). This analysis is performed voxel-wise. In this method, infarct growth corresponds to the volume of signal changes on DWI_2_ that does not overlap with that on co-registered DWI_1_ (36).

An automated three-dimensional rigid registration is used for MR images processing (FMRIB's Linear Image Registration Tool; FLIRT, V5.5) (37). Follow-up (24 h) DWI images are co-registered to pre-treatment DWI images, chosen as the target space. Registration quality is visually inspected for all cases. DWI lesions are segmented manually using our published methods (36, 38–40), based on DWI signal intensity (MANGO software, V3.1.1, Research imaging Institute, UTHSCSA). Initial and follow-up DWI lesions are outlined according to their maximal visual extent after careful adjustment of the window level. Segmented DWI lesions are transferred onto Apparent Diffusion Coefficient (ADC) maps to exclude any areas of T2 shine-through effect, and include areas of decreased ADC with subtle DWI signal changes. Segmented follow-up DWI lesions are transferred onto pre-treatment DWI maps to ensure a full coverage of brain tissue.

Infarct growth is also systematically estimated using the subtraction method, as ancillary assessment: in this method, the baseline DWI lesion volume is simply subtracted from the DWI lesion volume measured on follow-up MRI. All imaging data are analyzed by a neuroradiologist unaware of treatment-group assignment.

#### Secondary outcomes

We defined two secondary clinical outcomes, a safety outcome and three subgroups analyses.

- NIHSS: a neurological exam (with NIHSS score) is assessed at the admission and at 7 days or discharge if earlier. The difference between NIHSS score at day 7 (or discharge if earlier) and on admission constitutes the first clinical outcome assessment.
- Modified Rankin scale (mRs): the mRs is a 7-point disability scale ranging from 0 (no symptom) to 6 (death).The mRs is estimated at 3 months by phone interview using a simplified and standardized questionnaire (33). Whenever possible, the patient will be interviewed directly. If the patient is unable to complete the interview, the interview will be conducted based on information from the relatives or the patient's general practitioner. The mRs at 3 months constitutes the second clinical outcome assessment.

- Safety outcome: safety variables, including major adverse effects (neurologic deterioration) and minor adverse effects (itching sensations, cutaneous rash, headaches, vertigo, nausea) are monitored during tDCS stimulation, and then again at day 1 and day 2.
- Subgroup analyses are planned according to the following stratifications: (i) patients with and without perfusion–diffusion mismatch, with hypoperfusion defined as *T*_max_ > 6 s, on admission MRI. The perfusion–diffusion mismatch is defined by the volumetric ratio between perfusion lesion (at *T*_max_ > 6 s) and diffusion lesion (ratio > 1.8). The mismatch as defined is a neuroradiological marker of the ischemic penumbra, the target of our protocol; (ii) patients with NIHSS admission score above vs. below the median NIHSS admission score; (iii) presence or absence of arterial recanalization measured by the thrombolysis in cerebral infarction scale (TICI) post-arteriography or by MR Angiography at day 1 (successful recanalization on arteriography is estimated by a TICI scale 2b or 3) and (iv) patients with and without initial large vessel occlusion. The patient population will be clearly described with respect to vessel occlusion (no vessel occlusion, otherwise site of occlusion) in further publication.

### Statistical analysis

Continuous variables are described as mean ± standard deviation (SD) or median [interquartile range (IQR)] and are compared using the *t*-test or Mann-Whitney U-test. Categorical variables are expressed as percentages and compared using the Pearson χ^2^-test or Fisher's exact test, as appropriate. A univariate analysis is expected and adjustment is scheduled according to the success or not of recanalization. Primary outcome is analyzed in intention-to-treat, safety outcomes in per-protocol.

No intermediate analysis is planned. All data for the primary outcome will be assessed at the end of the study. The inclusion of 50 patients is planned over 30 months. This sample will allow to evaluate the feasibility of tDCS in the very acute phase of stroke and should highlight a tendency which will be used in further studies to demonstrate a statistically significant infarct growth difference between the two groups.

The number of patients (*n* = 25 per group) was empirically chosen to match the recruitment capacity of our center during the study period, and because we believe it would provide a reliable estimate of the effect size of the intervention for further studies. This planned number of patients was not based on a formal sample size calculation and we are aware that the present pilot trial will most likely be underpowered to demonstrate the superiority of the intervention over the control group with regard to the primary outcome.

In order to inform sample size and power calculations, we have used unpublished data from 200 consecutive patients who underwent mechanical thrombectomy for acute ischemic stroke in our institution and for whom infarct growth between admission and 24 h follow-up imaging was estimated using the method described in section Primary Outcome (voxel-wise infarct growth on DWI). These data were used to confirm the log-normal distribution of infarct growth and to provide the expected median and SD for the control group of STICA, namely 16 and 48.8 ml, respectively. Using the method described by O'Keeffe et al. (41), and assuming a 30% relative reduction in median infarct growth in the treatment group, 177 patients per group would be needed (two-sided comparison of untransformed medians, alpha = 5%, beta = 20%). Based on this effect size, a sample size of 25 patients per group would yield a statistical power of 18%. Conversely, with 25 patients per group, the effect size that could be detected with a statistical power equal to 80% would be a 65% relative reduction in infarct growth. Such an effect size may seem overly optimistic but the main aim of this pilot study is to provide a reasonable estimate of infarct growth reduction with cathodal tDCS, in order to inform the sample size calculation for, and feasibility of a multicenter phase IIb or phase III superiority trial.

## Discussion

Improving functional outcome is a priority in the management of acute ischemic stroke. Penumbral salvage by early reperfusion results in proportional clinical recovery. A promising novel approach, in order to enlarge the volume of salvageable penumbra at reperfusion time, and in turn improve final outcome, is to “freeze” the penumbra until reperfusion occurs.

In the penumbra, where energetic supplies are critically reduced due to the arterial occlusion, CSDs propagate, neuron cells cannot restore ion homeostasis and resting membrane potential, and cell death eventually occurs unless perfusion is rapidly restored. Moreover, neuronal depolarizations cause glutamate and nitric oxide release, resulting in excito-toxicity and apoptosis (42). The excito-toxicity also enhances neuronal death in the ischemic penumbra (17).

Our study is based on the possibility to limit excitotoxicity and neuronal death in the ischemic penumbra by applying cathodal tDCS early after the arterial occlusion and until revascularization. Three studies conducted on stroke rodent models were promising for a neuroprotective effect of cathodal tDCS in this context. In the first (23), cathodal tDCS starting 30 min into 90 min transient proximal MCA occlusion, in 75 mice, was able to preserve cortical neurons from ischemic damage: a significant reduction of infarct volume by 37% was observed in the cathodal tDCS group relative to the placebo tDCS groups. A significant decrease in cortical glutamate was observed using MR spectroscopy in mice treated by cathodal tDCS. By contrast, early-applied anodal tDCS, which increases neuronal activity (21, 43) and, hence, might aggravate neuronal oxygen deprivation in ischaemic conditions, mildly increased lesion volume (13, 23). In the second study (24), 36 rats underwent permanent MCA occlusion and were randomized in three groups: cathodal tDCS administered for 4 h or for 6 h, and sham-tDCS. The neuroprotective effect of cathodal tDCS was ascertained by a 20% reduction in infarct volume in the 4 h cathodal tDCS group and 30% in the 6 h cathodal tDCS group. Moreover, PIDs were recorded using a gold coated miniature screw inserted in the skull overlying the infarcted hemisphere. Cathodal stimulation reduced the number of depolarizations. Infarct volume correlated with the number of PIDs. In the third study (25), cathodal tDCS resulted in mild reductions (~25%) in infarct volume after branch permanent MCA occlusion.

tDCS is a well-tolerated neuromodulation technique which is increasingly used in neurological and psychiatric disorders (18, 44–54). However, it has not been used so far in the context of hyper-acute human ischemic stroke. We chose the tDCS parameters based on previous clinical studies. In most tDCS studies, current density varied between 0.029 and 0.08 mA/cm^2^ without serious adverse events (18).

In human studies cathodal-tDCS with current density of 0.029 mA/cm^2^ were shown to inhibit the sensorimotor cortices (55, 56). For our protocol, we chose a current density of 0.057 mA/cm^2^ which is both known to be safe in humans and higher than the current density known to inhibit sensorimotor cortices. The duration of excitability changes induced by tDCS depends on stimulation duration (57). In human studies, stimulation durations range from 3 to 40 min with iterative sessions (18). Based on the available preclinical stroke studies (23, 24), we chose a stimulation duration of 20 min beginning as soon as possible after stroke onset, with iterative sessions as follows: 20 min per hour over 6 h. The tDCS electrode sites (C3 or C4 on 10–20 system and contralateral supra-orbital area) were also chosen according to previous clinical studies.

Some tDCS studies have used one scalp electrode and one extra-cephalic electrode, located on the shoulder. However, with this montage, the current would have negative effects on deep structures such as the brainstem, with potential effects on autonomic functions. To avoid this risk, a cephalic tDCS montage is now recommended (58).

We chose to restrain inclusion criteria to patients with MCA stroke, i.e., the most common stroke subtype. The somatosensory area is frequently targeted in tDCS protocols. As we assumed that tDCS would freeze the penumbra area as an adjunct to revascularization therapies, we chose to restrict the recruitment to patients with acute ischemic stroke eligible to revascularization procedures.

We chose to evaluate infarct growth at 24 h from stroke onset with MRI. This time point may be discussed because infarct volume may not be completely stabilized by 24 h from stroke onset. Moreover, in the DEFUSE 3 trial, with a selected population of “slow ischemic growth” (also called “slow progressors”), imaging assessment at 24 h did not show difference between groups, contrary to clinical benefit. However, vasogenic edema usually occurs in the first week and can artificially inflate lesion volume. Previous studies demonstrated that 24 h evaluation of infarct volume by non-contrast computed tomodensitometry is as well correlated to 3 month-mRS than is 1 week evaluation (59, 60). There is currently no consensus about the best time-point to evaluate infarct growth, and future stroke studies are needed to address this issue.

Despite a rigorous study protocol, there are potential drawbacks in our study. The first is the limited sample. Thus, our study will not have the statistical power to demonstrate a significant reduction in infarct growth in the cathodal tDCS group. Although limiting the number of participants is necessary in a pilot study for safety concerns, this also necessarily limits the power of efficacy assessment. If the results of our study are promising, they will be useful for the design of a phase III clinical trial with more participants. Moreover, the rationale of our study is that tDCS may act on excitotoxicity pathways in the penumbra area, blocking CSDs and PIDs until reperfusion takes place. It should however be kept in mind that the phenomenon of PIDs after stroke has only been recorded in man in malignant MCA infarction, by means of electrocorticography (17, 61–63). There is no non-invasive tool allowing the identification of CSDs and PIDs during tDCS.

In summary, STICA is underpinned by the potential of cathodal tDCS to limit excitotoxicity and ischemic damage in acute human MCA stroke, as an adjunct to standard-of-care revascularization strategies. This pilot, randomized, double-blind placebo-controlled trial will recruit 50 patients and follow them over 3 months, assessing tDCS tolerance, infarct growth, and functional outcome. Depending on the results of this pilot study, a subsequent, larger study may be planned.

## Author contributions

EP-R: bibliography research, acquisition, analysis, and interpretation of data, wrote the first draft of the manuscript. DC, WB, GT, AM, NM, PS, and CO: acquisition of data, critical revision of the manuscript, read, and approved the submitted version. J-CB: bibliography research, clinical revision of the manuscript, read, and approved the submitted version. J-LM: study design and concept, critical revision of the manuscript, read, and approved the submitted version. MG: study design and concept, acquisition of data, critical revision of the manuscript, read, and approved the submitted version.

### Conflict of interest statement

The authors declare that the research was conducted in the absence of any commercial or financial relationships that could be construed as a potential conflict of interest.

## Abbreviations

CSD: cortical spreading depression
DWI: diffusion weighted imaging
EVT: endovascular thrombectomy
IA: intra-arterial
IG: infarct growth
IQR: interquartile range
MCA: middle cerebral artery
mRs: modified Rankin scale
NIHSS: National Institutes of Health Stroke Scale
PIDs: peri-infarct depolarizations
PWI: Perfusion-Weighted-Imaging
rtPA: recombinant tissue plasminogen activator
SD: standard deviation
tDCS: Transcranial direct current stimulation
TICI: thrombolysis in cerebral infarction scale.
